# Computational Characterization of Bidentate P-Donor Ligands: Direct Comparison to Tolman’s Electronic Parameters

**DOI:** 10.3390/molecules23123176

**Published:** 2018-12-01

**Authors:** Tímea R. Kégl, Noémi Pálinkás, László Kollár, Tamás Kégl

**Affiliations:** 1 Department of Inorganic Chemistry and MTA-PTE Research Group for Selective Chemical Syntheses, University of Pécs, H-7622 Pécs, Hungary; timea.kegl@gmail.com (T.R.K.); kollar@gamma.ttk.pte.hu (L.K.); 2 Department of Inorganic Chemistry, University of Pécs, H-7622 Pécs, Hungary; noemi.palinkas@gmail.com

**Keywords:** diphosphines, electronic parameters, DFT, QTAIM

## Abstract

The applicability of two types of transition-metal carbonyl complexes as appropriate candidates for computationally derived Tolman’s ligand electronic parameters were examined with density functional theory (DFT) calculations employing the B97D3 functional. Both Pd^(0)^L_2_(CO) and HRh^(I)^L_2_(CO) complexes correlated well with the experimental Tolman Electronic Parameter scale. For direct comparison of the electronic effects of diphosphines with those of monophosphines, the palladium-containing system is recommended. The *trans* influence of various phosphines did not show a major difference, but the decrease of the H-Rh-P angle from linear can cause a significant change.

## 1. Introduction

Phosphines, phosphites, and other P-donor ligands are of vital importance in the wide range of reactions catalyzed by transition-metal (TM) compounds [1,2,3]. Changing the coordinated ligands is an effective strategy for modifying the properties of transition metal containing catalysts. The chemical properties of transition-metal complexes, such as their activity in various catalytic reactions, are principally determined by the steric and electronic properties of the ligands bound to the metal center [4,5,6,7,8]. Their structural variation opens the possibility to tune catalytic activity, as well as chemo-, regio-, and enantioselectivity [9,10]. It has long been known that variation of the substituents can cause changes upon coordination in the behavior of the ligands as well as their TM complexes. Information about the nature of the transition metal–phosphorus bond and its influence on other bonds in the molecule is crucial for the characterization of catalytically active compounds and for the fine-tuning their properties in order to develop more efficient catalysts.

In the 1960s, Strohmeier et al. investigated the σ-donor ability and π-acceptor strength of various ligand classes (nitriles, isonitriles, sulfoxides, phosphines). The phosphines were further divided into four categories designated from I to IV, classified by the frequency regions of 1927–1996, 1946–1982, 2045–2101, and 2060–2110 cm^-1^ for complexes C_5_H_5_Mn(CO)_2_L, C_5_H_5_V(CO)_3_L, Fe(CO)_4_L, and Ni(CO)_3_L, respectively, and sorted by wavenumber into increasing order [11]. These classes constitute the basis for Tolman’s famous formula.

In the 1970s, Tolman reported the A${}_{1}$ carbonyl-stretching frequencies of NiL(CO)_3_ complexes, with monodentate P-donor ligands (L = PR${}^{1}$R${}^{2}$R${}^{3}$) [2,12]. The electronic effects of 70 phosphorus-containing ligands were compared. It was found that a contribution could be assigned to each substituent on phosphorus, and the sum of those contributions were equal to the entire influence of the ligand on ν(CO). This contribution was designated as $\chi_{i}$ (cm${}^{-1}$), and the most basic ligand (tri-*t*-butylphosphine) was chosen as reference on the Tolman electronic parameter (TEP) scale recognizing that PBu${}_{3}^{t}$ is the best σ-donor and the worst π-acceptor ligand (Equation (1)). The NiL(CO)_3_ scale, that is, the TEP, can be correlated with Strohmeier’s CpMnL(CO)_2_ system [7,13] (Equation (2)).

(1) $$\nu {\left(\mathrm{CO}\right)}_{Ni}=2056.1+\Sigma \chi_{j}$$

(2) $$\nu {\left(\mathrm{CO}\right)}_{Ni}=0.711\cdot \nu {\left(\mathrm{CO}\right)}_{Mn}+692\;\;\;\left({\mathrm{cm}}^{-1}\right)\;R\;=\;0.970$$

Chelating ligands, such as diphosphines, however, cannot be described within the framework of the TEP scale. Crabtree et al. suggested more suitable model complexes for chelating ligands, for example, MoL_2_(CO)_4_ (L${}_{2}$: bidentate phosphine ligand or two monodentate phosphines) [14]. They compared 11 phosphorus ligands of various types and correlated to the existing TEP scale using Equation (3). Thus, Crabtree proved Tolman’s statement that the choice of transition-metal carbonyl system is arbitrary and interchangable.

(3) $$\nu {\left(\mathrm{CO}\right)}_{Ni}=0.593\cdot \nu {\left(\mathrm{CO}\right)}_{Mo}+871\;R\;=\;0.996$$

In a recent study comparing the Rh–Vaska complexes and NiL(CO)_3_, Otto and Roodt established a simple quadratic equation [15]. Later, the relationship between the carbonyl-stretching frequencies of the two reference complexes were revised and, by the basicities of the ligands, the ν(CO) range was divided into two sections [16]. The slope for the more basic phosphines showed a difference to those of less basicity.

Arsines and stibines were investigated in order to determine their electronic effects as well [16]. The contributions to the electronic effect of As and Sb, relative to P, were determined, and an additional term compared to Equation (1) was suggested addressing the effect of the donor atom (C${}_{L}$; L = P, Sb or As). (Equation (4), C${}_{P}$ = 0)

(4) $$\nu {\left(\mathrm{CO}\right)}_{Ni}=2056.1+\Sigma \chi_{j}+C_{L}$$

In the past three decades several attempts have been made for invoking theoretical methods to characterize the donor and acceptor properties of phosphines and other, mainly P-donor, ligands. These methods can be divided into two categories. The first approximation deals only with the isolated ligand, focusing on its electronic and steric properties, while neglecting the influence of the metal-containing fragment. As a prominent example, the molecular electrostatic potential at the lone pair of the phosphorus atom should be mentioned, which correlates reasonably well with the TEP scale, according to Suresh and Koga [17]. Moreover, the method known as quantitative analysis of ligand effects (QALE) relies on experimental data of known ligands and provides the resolution of net donating ability into QALE parameters [18,19]. It should be noted that the pnictogen interactions, as important secondary interactions, of a wide variety of P-donor ligands were studied as well [20,21,22]. The second category uses approaches that focus on the entire transition-metal complex, thereby including the possibility to scrutinize ligand–ligand effects as well [23]. Various electronic structure methods, such as Extended Transition State theory combined with Natural Orbitals of Chemical Valence (ETS-NOCV) [24,25,26,27] and Quantum Theory of Atoms in Molecules (QTAIM) [26] have been employed as well.

Crabtree and coworkers investigated the CEP, that is, the computationally derived ligand electronic parameter using the NiL(CO)_3_ model complex with various neutral, cationic, and anionic ligands [28]. They found that the CEP correlated well with the Tolman electronic parameter and, for certain anionic ligands, even with the Hammett *meta* substituent constant.

Although the TEP scale achieved a widely accepted status, the limitations of this approximation should be mentioned as well. The coordinating properties of a ligand are governed together by its σ-donor and π-acceptor abilities, whereas TEP (or CEP) only gives the *net* donor strength. Moreover, through-space ligand interactions may affect the carbonyl-stretching frequencies as reported by Sierra and coworkers for manganese half-sandwich complexes [29]. The Tolman electronic parameters failed for linear Au–carbonyl complexes [30].

The goal of this computational study is to establish a relationship between the ligand electronic effects of late transition-metal–diphosphine complexes with the Tolman’s scale, that is, the totalsymmetric carbonyl-stretching frequencies of Ni(CO)_3_L complexes, thereby providing a direct comparison of the electronic effects of diphosphines and monophosphines. By the selection of appropriate ligands, the set of ligands employed by Crabtree [14] and Tolman [12] has been adopted, that is, PEt_3_ (**1**), PEt_2_Ph (**2**), PMe_3_ (**3**), PMe_2_Ph (**4**), PPh_3_ (**5**), P(OMe)_3_ (**6**), P(OPh)_3_ (**7**), PCl_2_(OEt) (**8**), PCl_3_ (**9**), PF_3_ (**10**), and PF_2_(CF_3_) (**11**).

**Figure 1:**
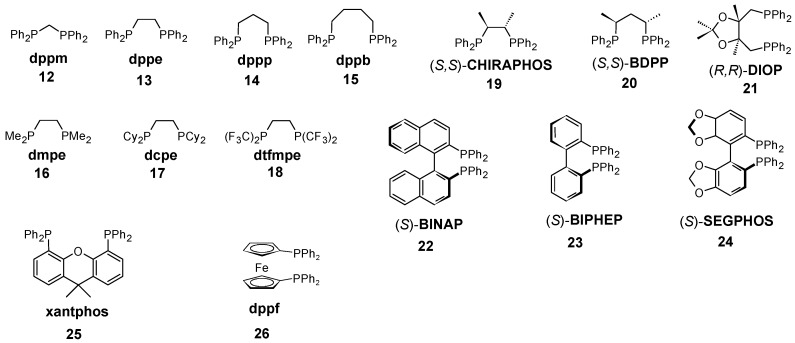
Chelating phosphines considered in this study.

The secondary objective of the study is to find a dependence of the trans influence upon the bite angle of the diphosphines by scrutinizing the Rh–H bond distance, the rhodium–hydride stretching frequencies as well as quantum chemical descriptors within the framework of the QTAIM methodology. Those diphosphines that are involved in this study are depicted in Figure 1. As test complexes, the Pd^(0)^L_2_(CO) and the HRh^(I)^L_2_(CO) complexes were selected. The former is supposed to be sterically less strained in comparison to the Mo(CO)_4_L_2_ complexes with a natural bite angle of about 90${}^{\circ }$, thereby reducing the possibility of coupling steric and electronic effects and through-space interactions, whereas the rhodium-containing species are of high importance as the potential catalysts of Rh-catalyzed hydroformylation. It should be noted that only the theoretical treatment of the selected test complexes is possible because of their high reactivity, rendering them unsuitable for the experimental spectroscopical measurements.

## 2. Computational Details

All the structures were optimized without symmetry constraints with tight convergence criteria using the program ORCA 4.0.1 [31] with the exchange and correlation functionals developed by Grimme [32] containing the D3 empirical dispersion correction with Becke and Johnson damping [33]. For palladium and rhodium atoms the def2-TZVP basis set [34]. was employed with the respective pseudopotentials [34]. For the other atoms, the def2-SVP basis set [34] was used. The B97D3 functional was selected for the calculations involving diphosphines as it gave slightly better linear correlations for monophosphines as compared to other GGA (BP86 [35,36], PBEPBE [37]) and meta-GGA (TPSS [38], and M06L [39]) functionals (*vide infra*).

For the complexes containing conformationally flexible ligands, conformational analyses have been performed in a similar manner reported earlier [26], and the lowest energy species were considered. QTAIM analyses of the wave function [40] were carried out with AIMAll software [41]. Topological analyses of the Electron Localization Function (ELF) were completed using the Topmod09 package [42]. For electronic structure calculations, the B97D3/def2-TZVP level of theory was employed on the equilibrium geometries obtained by the B97D3 calculations with the mixed (def2-TZVP/def2-SVP) basis set.

## 3. Results and Discussion

**Figure 2:**
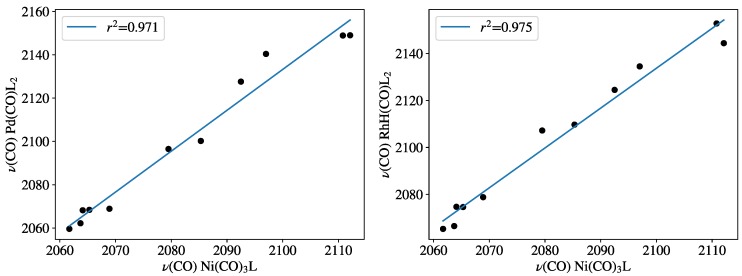
Carbonyl-stretching frequencies of PdL_2_(CO) and HRhL_2_(CO) against Tolman’s parameters at the B97D3 level. Frequency values are given in Table 1.

In Table 1, the experimental nickel ν(CO) parameters, as well as the computed palladium, rhodium ν(CO) parameters were collected for various phosphorus ligands, the same ligands that had also been used by Crabtree et al. In Figure 2, the 11 calculated (B97D3) carbonyl-stretching frequencies of PdL_2_(CO) and HRhL_2_(CO) training sets are plotted against the experimental ν(CO) frequencies for NiL(CO)_3_ [2,12]. The high values of the correlation coefficients r${}^{2}$ = 0.971 and r${}^{2}$ = 0.975, obtained for PdL_2_(CO) and HRhL_2_(CO), respectively, shows that the computed ν(CO) for these systems (CEP parameters) have reasonable linear correlation with the experimental NiL(CO)_3_ν(CO) values (TEP) according to Equations (5) and (6):

(5) $$\nu {\left(\mathrm{CO}\right)}_{\mathrm{Ni},\exp}={\mathrm{CEP}}_{\mathrm{Pd}}=0.5144\cdot \nu {\left(\mathrm{CO}\right)}_{{\mathrm{PdL}}_{2}\left(\mathrm{CO}\right)}+1002.2$$

(6) $$\nu {\left(\mathrm{CO}\right)}_{\mathrm{Ni},\exp}={\mathrm{CEP}}_{\mathrm{Rh}}=0.5744\cdot \nu {\left(\mathrm{CO}\right)}_{{\mathrm{HRhL}}_{2}\left(\mathrm{CO}\right)}+874.0$$

**Figure 3:**
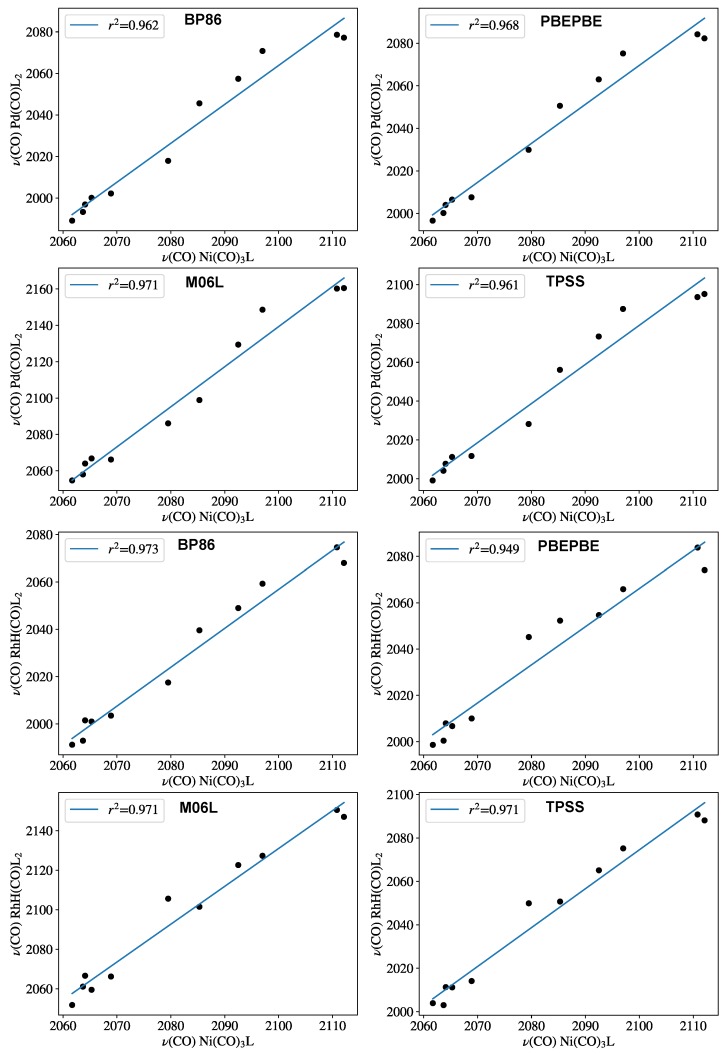
Carbonyl-stretching frequencies of PdL_2_(CO) (top row) and HRhL_2_(CO) against Tolman’s parameter computed with various DFT functionals. Frequency values are given in Table 1.

To clarify the role of the theoretical method employed for obtaining CEP parameters, the training set was recomputed with various GGA and meta-GGA functionals (Figure 3). The B97D3 results were compared with those obtained with BP86 [35,36], PBEPBE [37], TPSS [38], and M06L [39] functionals for both the palladium and the rhodium complexes. The $r^{2}$ correlation coefficients remained within quite a narrow range, as the worst correlation was found to be $r^{2}=0.949$ for the PBEPBE functional in the case of the Rh–hydride model systems. For both complexes, the B97D3 functional proved to be slightly superior; therefore, it was chosen for describing the chelating ligands as well.

The chelating ligands shown in Table 2 were selected to address various geometrical as well as electronic parameters. Ligands **12**–**15** were used to illustrate the influence of the P-M-P bite angle while ligands **16**–**18** show the effect of the substituents on the phosphorus with ethanediphos backbone. The chiral ligands **19**–**21** reveal the substituent effects on the backbone. To address the structural changes of ligands with axial chirality **22**–**24** were selected. Finally, **25** and **26** represent ligands possessing rigid structure with wide bite angles.

The CEP determined on two different types of carbonyl complexes reveals a remarkable difference (near 10 cm${}^{-1}$) for the narrow bite-angle case **12**. The increase of the bite angle by going in the direction of dppm → dppe → dppp → dppb also caused a decrease in ν(CO) for the palladium and an increase in ν(CO) for the rhodium complexes. The replacement of the phenyl substituent in dppe to the electron-withdrawing trifluoromethyl group resulted in a similar blue-shift for the two systems. On the other hand, the presence of electron-donating substituents, such as methyl or cyclohexyl, on the phosphorus results in the expected red-shift in the palladium carbonyls and a mixed effect for the Rh carbonyls as, in HRh(CO)(dmpe), virtually no change occurs in the carbonyl-stretching frequency. When the electron-donating methyl groups are introduced in the chiral backbone, such as for the ligand CHIRAPHOS, a slight blue-shift occurs for HRh(**19**)(CO), whereas an almost negligible red-shift takes place for Pd(**19**)(CO).

The change or the neglect of the condensed ring in the ligands with axial chirality has practically no effect on ν(CO). Moreover, the predicted CEP is almost identical for both model systems. This difference is also minor (below 2 cm${}^{-1}$) for the xantphos and dppf ligands. Not surprisingly, in the three-coordinate palladium systems, all the P-M-P bite angles were somewhat larger than in the HRh(LL)(CO) complexes, except for the dppm ligand.

The trans influence in the four-coordinate, distorted square-planar HRhL_2_(CO) complexes is formally based on a $n_{P}\to \sigma_{\mathrm{RhH}}^{*}$ donor–acceptor interaction. Here, it is followed by the metal–hydrogen stretching frequency (ν(RhH)), the Rh–H distance, and two QTAIM parameters. Both the electron density at the bond critical point ($\rho_{\mathrm{BCP}}$) and the δ(Rh,H) delocalization index, which provides the number of electron pairs delocalized between the atomic basins of Rh and H [43], were somewhat proportional with the bond strength. The delocalization index is, to some extent, related to formal bond orders for an equally shared pair between two atoms in a polyatomic molecule.

According to the $\rho_{\mathrm{BCP}}$(RhH) values in Table 3 the difference in electronic effects caused by the P-ligand trans to the hydride is rather subtle. As the trend in the delocalization indices is quite the opposite compared to that obtained from the geometrical parameters, it is likely that the electron sharing between atomic basins did not reflect the overall bond strengths in these cases.

The ν(RhH) frequencies, however, tend to follow the computed electronic parameters, as the largest red-shift occurs for the more electron donating ligands and the frequencies are increased for the electron withdrawing ones. Thus, the trans influence increases in the order of the substituent donor strength.

**Figure 4:**
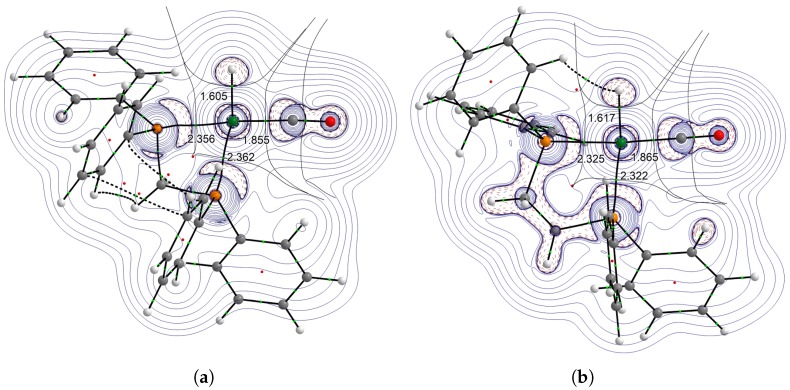
Laplacian ($\nabla^{2}\rho \left(r\right)$) of the electron density of complexes HRh(dppm)(CO) (**a**) and HRh(dppe)(CO) (**b**). Charge concentration regions ($\nabla^{2}\rho \left(r\right)<0$) are designated with dashed lines.

To address the effect of the P–Rh–P bite angle upon the trans influence, the structures and the Laplacians of the electron density were compared and are illustrated in Figure 4. The structural distortion caused by the dppmligand resulted in distortion in the electron-density distribution aswell. The stronger bond in the dppm complex, as compared to the HRh(dppe)(CO) complex, shows that even when the basicities of the phosphorus atoms of the standalone dppm and dppe ligands should be very similar, the slight decrease of the H–Rh–P angle causes a notable decrease in the trans influence. It should be noted that, in the dppm complex, a red-shift can be observed in ν(CO) in comparison to that of the dppe complex, which may be explained with the less preferred orientation to a $n_{P}\to \pi_{\mathrm{CO}}^{*}$ orbital interaction.

It was found previously that, among QTAIM parameters, CO delocalization index gave good performance, whereas the electron density of the CO bond critical point gives moderate linear correlation with the computed carbonyl symmetric stretching frequencies in Ni-tricarbonyl phosphine complexes [26]. To the best of our knowledge, the relationship between the electron localization function (ELF) and the stretching frequencies in transition-metal carbonyl complexes is unexplored.

The ELF, denoted as η(r) introduced by Becke and Edgecombe [35] and reinterpreted by Savin et al. [44] using the mathematical scheme from Thom’s catastrophe theory [45] is based on the conditional pair probability function. It takes values in a range 0≦η≦1 between 0 and 1, where values close to 1 are located in regions of space with strong electron localization, whereas values close to 0 correspond to electron delocalization, and η = 0.5 is the value that would be expected for a homogeneous electron gas. It is worth mentioning that Putz [46,47] proposed general formulations for the ELF on the basis of the path integral formulation of Markovian open systems.

The electronic effect of the monodentate ligands was followed by the population of the V(C,O) and V(Rh,H) disynaptic basins. The population of an ELF basin is defined according to Equation (7).

(7) $$\bar{N}\left(\Omega_{i}\right)=\int_{\Omega_{i}}\rho \left(r\right)dr$$

**Figure 5:**
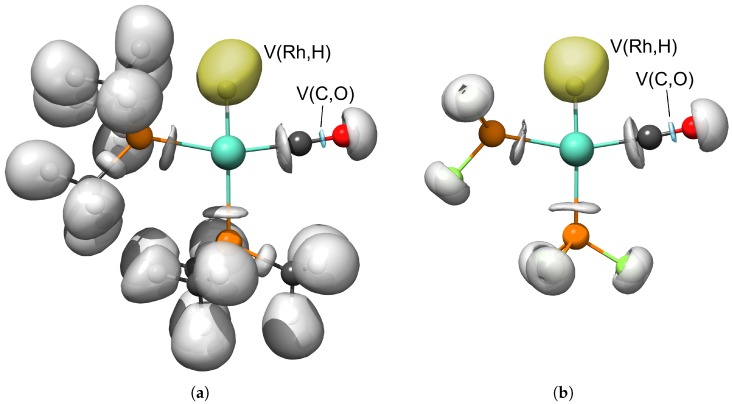
Selected electron localization function (ELF) valence basins for complexes HRh(PMe_3_)_2_(CO) (**a**) and HRh(PF_3_)_2_(CO) (**b**) at isovalue of η = 0.84. Yellow and blue envelopes designate the disynaptic V(Rh,H) and V(C,O) basins, respectively.

The ELF basins, which were the subject of our study, are depicted for complexes HRh(PMe_3_)_2_(CO) and HRh(PF_3_)_2_(CO) in Figure 5. The spacial distribution of the V(Rh,H) basin resembles those representing the H–C bonds, that is, it is localized around the hydrogen and not along the H–Rh bond path. On the other hand, the V(C,O) basins are situated in the middle between the carbon and oxygen of the carbonyl ligands.

**Figure 6:**
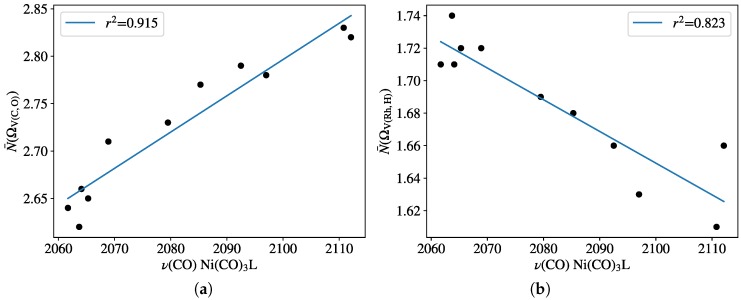
Population of ELF basins (**a**) V(C,O) and (**b**) V(Rh,H) for complexes HRhL_2_(CO) against Tolman’s parameters at the B97D3 level. Frequency values are given in Table 1.

The ELF was computed for all the HRh(monophosphine)_2_(CO) type of complexes. The basin population associated with the C≡O bond revealed fairly good linear correlation (r^2^ = 0.915) with the experimental TEP parameters (Figure 6a). The extrema of the $\bar{N}\left(\Omega \right)$ values are between 2.62 (for ligand PEt_2_Ph) and 2.83 (for PF_3_), indicating the somewhat depleted triple-bond character of the bound CO. The population change of the Rh–hydride bond, however, shows moderate linear correlation (r^2^ = 0.823) with TEP; only showing that the trans influence generally increases with the greater donor strength of the P-donor ligand.

## 4. Conclusions

In the present study, two classes of model compounds were scrutinized to check their applicability as appropriate candidates for computationally derived ligand electronic parameters (CEPs) for bidentate P-donor ligands employing the B97D3/def2-TZVP(def2-SVP) level of theory. The results of this work can be summarized as follows:

- For both model systems, no major difference in accuracy can be expected for various DFT methods in terms of the linear regression coefficient.
- For monophosphines, both Pd^(0)^L_2_(CO) and HRh^(I)^L_2_(CO) complexes showed high linearity with the experimental TEP scale.
- For monophosphines, the population of the disynaptic ELF basin V(C,O), associated with the carbonyl ligand, showed good linearity with the experimental TEP scale.
- For diphosphine-containing systems, the bite angle effect showed the opposite trend, with PdL_2_(CO) complexes revealing a decrease in ν(CO) by the increase of bite angle.
- In the case of the Pd complexes, the change of substituents on phosphorus caused a change in ν(CO) being consistent with the donor character of the substituent.
- For diphosphines possessing axial chirality, the nature of the condensed ring does not alter the electronic parameter of the ligand.
- The trans effect estimated in the HRhL_2_(CO) complexes is very sensitive to the H–Rh–P angle.

## Figures and Tables

**Table 1:** Experimental CO-stretching frequencies (Reference [12]) for NiL(CO)_3_, and computed CO-stretching frequencies for PdL_2_(CO) and HRhL_2_(CO) complexes at the B97D3 level of theory. Carbonyl-stretching frequencies are given in cm${}^{-1}$.

Ligand	ν(CO)${}_{\mathrm{NiL}{\left(\mathrm{CO}\right)}_{3}}$	ν(CO)${}_{{\mathrm{PdL}}_{2}(\mathrm{CO})}$	ν(CO)${}_{{\mathrm{HRhL}}_{2}(\mathrm{CO})}$
PEt_3_ (**1**)	2061.7	2059.6	2065.3
PEt_2_Ph (**2**)	2063.7	2062.2	2066.5
PMe_3_ (**3**)	2064.1	2068.2	2074.7
PMe_2_Ph (**4**)	2065.3	2068.4	2074.6
PPh_3_ (**5**)	2068.9	2068.9	2078.8
P(OMe)_3_ (**6**)	2079.5	2096.5	2107.2
P(OPh)_3_ (**7**)	2085.3	2100.2	2109.7
PCl_2_(OEt) (**8**)	2092.5	2127.6	2124.5
PCl_3_ (**9**)	2097.0	2140.4	2134.5
PF_3_ (**10**)	2110.8	2148.9	2152.8
P(CF_3_)F_2_ (**11**)	2112.1	2149.0	2144.4

**Table 2:** Computed CO-stretching frequencies and P-M-P bite angles (*θ*_PPdP_ and *θ*_PRhP_, in degrees) for diphosphine-containing complexes PdL_2_(CO), HRhL_2_(CO), and their respective Computed Tolman Parameters (CEPs), determined according to Equations (5) and (6). Vibrational frequencies are in cm${}^{-1}$.

Ligand	ν(CO)${}_{{\mathrm{PdL}}_{2}(\mathrm{CO})}$	CEP${}_{\mathrm{Pd}}$	*θ*_PPdP_	ν(CO)${}_{{\mathrm{HRhL}}_{2}(\mathrm{CO})}$	CEP${}_{\mathrm{Rh}}$	*θ*_PRhP_
dppm (**12**)	2080.8	2072.6	69.8	2069.8	2062.9	71.0
dppe (**13**)	2076.1	2070.1	85.1	2071.9	2064.1	84.7
dppp (**14**)	2068.4	2066.2	90.5	2074.6	2065.7	88.7
dppb (**15**)	2064.5	2064.2	96.2	2079.0	2068.2	94.6
dmpe (**16**)	2073.5	2068.8	86.0	2075.1	2065.9	85.5
dcpe (**17**)	2058.6	2061.1	86.2	2060.6	2057.6	85.3
dtfmpe (**18**)	2123.1	2094.3	86.3	2125.8	2095.1	84.9
CHIRAPHOS (**19**)	2074.3	2069.2	85.1	2076.3	2066.6	84.6
BDPP (**20**)	2067.7	2065.8	89.0	2071.2	2063.7	87.7
DIOP (**21**)	2069.6	2066.8	101.5	2077.4	2067.3	95.5
BINAP (**22**)	2069.0	2066.5	93.7	2075.9	2066.4	92.8
BIPHEP (**23**)	2069.6	2066.8	96.4	2076.8	2066.9	93.8
SEGPHOS (**24**)	2067.5	2065.7	94.3	2073.9	2065.2	92.8
xantphos (**25**)	2065.0	2064.4	105.9	2070.1	2063.1	102.8
dppf (**26**)	2069.3	2066.6	103.6	2078.6	2067.9	97.8

**Table 3:** Computed Rh-stretching frequencies, Rh–H bond lengths (in Å), P–Rh–P bond angles (in degrees), delocalization indices, and electron densities at bond critical points for HRhL_2_(CO) complexes.

Ligand	ν(RhH)	r${}_{\mathrm{RhH}}$	$\theta_{\mathrm{PRhP}}$	δ(Rh,H)	$\rho_{\mathrm{BCP}}$(RhH)
PEt_3_ (**1**)	1920.8	1.618	100.4	0.858	0.131
PEt_2_Ph (**2**)	1931.6	1.615	95.6	0.873	0.132
PMe_3_ (**3**)	1927.3	1.618	100.9	0.860	0.131
PMe_2_Ph (**4**)	1948.3	1.611	98.4	0.876	0.133
PPh_3_ (**5**)	1964.7	1.606	102.6	0.849	0.135
P(OMe)_3_ (**6**)	1949.0	1.618	100.7	0.844	0.132
P(OPh)_3_ (**7**)	1984.5	1.607	106.9	0.831	0.135
PCl_2_(OEt) (**8**)	2014.5	1.598	96.4	0.866	0.139
PCl_3_ (**9**)	1993.0	1.602	101.2	0.837	0.135
PF_3_ (**10**)	1970.6	1.614	99.9	0.841	0.134
P(CF_3_)F_2_ (**11**)	1967.5	1.617	100.2	0.829	0.133

## Supplementary Materials

The following are available online. Cartesian coordinates of palladium-diphosphine complexes occuring in this study.
