# Supplementary material for: Computational Characterization of Bidentate P-Donor Ligands: Direct Comparison to Tolman’s Electronic Parameters
Source: Molecules. 2018 Dec 1;23(12):3176. doi: 10.3390/molecules23123176 (PMC6321482; doi:10.3390/molecules23123176)
Supplement: Supplementary file 1 [file molecules-23-03176-s001.pdf]

Computational Characterization of Bidentate P-donor Ligands: Direct Comparison to Tolman's Electronic Parameters

Tímea Kégl, Noémi Pálinkás, László Kollár, Tamás Kégl

Cartesian coordinates of palladium-diphosphine complexes occuring in this study

All energy values are given in a.u.

Pd(dppe)(CO) (Pd(**13**)(CO))

Pd	15.19792	-27.52594	-16.77965
P	15.81869	-26.31602	-18.73061
P	16.14016	-25.69271	-15.59002
C	15.43896	-24.81242	-14.13540
C	15.05119	-25.61455	-13.03987
C	15.23890	-23.41881	-14.06725
C	14.49612	-25.03331	-11.89379
C	14.66579	-22.84033	-12.92249
C	14.29813	-23.64307	-11.83323
H	15.18215	-26.70290	-13.10119
H	15.52982	-22.77135	-14.90114
H	14.20358	-25.66749	-11.04835
H	14.50934	-21.75525	-12.88362
H	13.85210	-23.18906	-10.94017
C	14.40479	-25.32520	-19.39087
C	14.40679	-24.77426	-20.68782
C	13.30241	-25.08978	-18.54217
C	13.32544	-23.99463	-21.12517
C	12.22915	-24.29871	-18.97766
C	12.23772	-23.75156	-20.27046
H	15.25262	-24.96358	-21.35907
H	13.28935	-25.54511	-17.54276
H	13.33135	-23.57586	-22.13862
H	11.37886	-24.11979	-18.30863
H	11.39298	-23.14288	-20.61512
C	17.87772	-26.04169	-15.06517
C	18.52141	-27.18450	-15.58091
C	18.58411	-25.18073	-14.20145
C	19.85647	-27.45595	-15.24674
C	19.91560	-25.45943	-13.86212
C	20.55467	-26.59554	-14.38525
H	17.95890	-27.86398	-16.23589
H	18.08733	-24.29508	-13.78758
H	20.34780	-28.34854	-15.65157
H	20.45700	-24.78777	-13.18453
H	21.59452	-26.81337	-14.11480
C	16.62267	-26.96985	-20.24926
C	17.75494	-26.39322	-20.85961
C	16.06106	-28.13780	-20.80952
C	18.31046	-26.97276	-22.01181
C	16.60744	-28.70389	-21.96789
C	17.73637	-28.12380	-22.57097
H	18.20956	-25.48755	-20.44651
H	15.19599	-28.60199	-20.31959
H	19.19442	-26.51885	-22.47507
H	16.15980	-29.60866	-22.39490
H	18.17007	-28.57191	-23.47226
C	16.96338	-24.93584	-18.15934
C	16.44131	-24.33089	-16.85143
H	17.95875	-25.39102	-18.00033
H	17.05813	-24.15889	-18.93844
H	15.47233	-23.82550	-17.02361
H	17.15194	-23.58554	-16.45073
C	14.36724	-29.19643	-16.35402
O	13.86517	-30.20979	-16.09128

Pd(dppp)(CO) (Pd(**14**)(CO))

Pd	13.68282	-26.92126	-16.33312
P	14.92323	-26.58011	-18.33721
P	15.01567	-25.39070	-15.14794
C	14.66165	-24.77781	-13.43946
C	13.36655	-24.96642	-12.92235
C	15.63449	-24.12505	-12.65607
C	13.04307	-24.49720	-11.64004
C	15.31020	-23.66054	-11.37377
C	14.01359	-23.84416	-10.86475
H	12.62392	-25.49572	-13.53356
H	16.65207	-23.99349	-13.04261
H	12.03309	-24.65210	-11.24205
H	16.07355	-23.16020	-10.76549
H	13.76327	-23.48564	-9.85950
C	13.88599	-26.33788	-19.85101
C	14.44737	-26.01123	-21.10411
C	12.48949	-26.47176	-19.73996
C	13.62413	-25.81739	-22.22091
C	11.66445	-26.27423	-20.85884
C	12.22976	-25.94515	-22.09912
H	15.53576	-25.92772	-21.21407
H	12.06335	-26.73687	-18.76433
H	14.07010	-25.56695	-23.19116
H	10.57744	-26.38105	-20.75887
H	11.58692	-25.79005	-22.97404
C	16.73014	-26.05698	-14.96244
C	17.91214	-25.37006	-15.29867
C	16.82693	-27.38895	-14.50049
C	19.16068	-26.00564	-15.19614
C	18.07259	-28.01759	-14.38486
C	19.24430	-27.32965	-14.74160
H	17.87414	-24.33494	-15.65366
H	15.90898	-27.93542	-14.25091
H	20.07072	-25.46187	-15.47607
H	18.12806	-29.05311	-14.03082
H	20.21902	-27.82691	-14.67028
C	16.19239	-27.80958	-18.85300
C	17.36785	-27.93631	-18.08013
C	15.97714	-28.70986	-19.91751
C	18.31950	-28.91690	-18.38624
C	16.93122	-29.69416	-20.21776
C	18.10782	-29.79797	-19.45926
H	17.53634	-27.27497	-17.22556

H	15.06159	-28.64027	-20.51431
H	19.22751	-28.99151	-17.77605
H	16.75307	-30.38305	-21.05259
H	18.85340	-30.56601	-19.69815
C	15.89390	-24.96748	-18.30720
C	15.21469	-23.77321	-16.07137
H	16.85805	-25.15319	-17.80529
H	16.13040	-24.67846	-19.34509
H	14.41017	-23.12841	-15.67482
H	16.16140	-23.28794	-15.76780
C	15.09230	-23.85317	-17.60631
H	15.41072	-22.87516	-18.01506
H	14.02417	-23.95830	-17.87812
C	12.10286	-27.93419	-15.97658
O	11.13556	-28.54236	-15.76066

Pd(dppf)(CO) (Pd(**26**)(CO))

Fe	16.23880	-28.76483	-10.95779
C	16.20245	-30.75813	-11.38877
C	17.31236	-28.35176	-9.26710
C	17.27276	-30.12645	-12.10080
C	14.96412	-30.12637	-11.77855
C	15.90819	-28.48902	-8.96531
C	17.46378	-27.28887	-10.21774
C	16.70944	-29.09976	-12.93061
C	15.28807	-29.09710	-12.73649
C	15.20214	-27.49800	-9.74295
C	16.16202	-26.75984	-10.50846
H	16.29930	-31.55742	-10.65203
H	18.11035	-28.97662	-8.86262
H	18.33535	-30.36065	-11.99989
H	18.40415	-26.96366	-10.66918
H	17.26974	-28.41377	-13.57111
H	14.57362	-28.41478	-13.20055
H	14.12049	-27.35548	-9.75910
H	15.93489	-25.96025	-11.21735
P	13.34921	-30.59719	-11.06544
P	15.06223	-29.69836	-7.88563
C	12.26326	-29.20147	-11.60556
C	11.91287	-29.00213	-12.95523
C	11.76974	-28.31522	-10.62769
C	11.10977	-27.91229	-13.32303
C	10.96832	-27.22427	-10.99656
C	10.64264	-27.01779	-12.34624
H	12.26117	-29.70794	-13.71828
H	12.00902	-28.50055	-9.57194
H	10.84079	-27.76479	-14.37609
H	10.58991	-26.54111	-10.22701
H	10.01387	-26.16738	-12.63697
C	12.80755	-31.95712	-12.19442
C	13.48757	-32.29630	-13.38006
C	11.65290	-32.67829	-11.82876
C	13.01152	-33.33811	-14.19174
C	11.17142	-33.70558	-12.64965
C	11.85177	-34.04013	-13.83249
H	14.39072	-31.74703	-13.66810
H	11.14429	-32.43165	-10.88805
H	13.54947	-33.59960	-15.11108
H	10.26761	-34.25533	-12.36095
H	11.48197	-34.85306	-14.46906
C	16.45574	-30.81896	-7.41478
C	16.45135	-32.14248	-7.89641
C	17.51368	-30.38838	-6.59018
C	17.50514	-33.01486	-7.58424
C	18.56436	-31.26238	-6.27481
C	18.56510	-32.57459	-6.77599
H	15.60133	-32.48698	-8.50104
H	17.50917	-29.36869	-6.18790
H	17.49154	-34.04330	-7.96400

H	19.38356	-30.92019	-5.63032
H	19.38540	-33.25791	-6.52487
C	14.81359	-28.71849	-6.33714
C	14.04043	-29.30650	-5.31470
C	15.34676	-27.42906	-6.14505
C	13.82302	-28.62404	-4.11121
C	15.11321	-26.74137	-4.94343
C	14.35617	-27.33771	-3.92390
H	13.60488	-30.29984	-5.47889
H	15.94302	-26.96157	-6.93641
H	13.22565	-29.09195	-3.31931
H	15.52574	-25.73497	-4.80474
H	14.17443	-26.79811	-2.98652
Pd	13.22462	-30.93266	-8.72549
C	11.97415	-31.97299	-7.71671
O	11.22252	-32.60408	-7.09491

Pd(xantphos)(CO) (Pd(**25**)(CO))

Pd	17.72605	-27.55667	-17.20821
P	16.32360	-26.73618	-18.94911
P	17.42272	-26.07255	-15.37176
C	18.37476	-24.50374	-15.58315
C	19.37382	-24.03640	-14.70896
C	18.15410	-23.77418	-16.76636
C	20.12092	-22.89576	-15.04020
C	18.88227	-22.62985	-17.12845
C	19.88389	-22.20218	-16.23861
H	19.57172	-24.57684	-13.77754
H	20.90081	-22.54301	-14.35511
H	20.48253	-21.31624	-16.47178
C	16.98114	-25.20937	-19.77181
C	17.41450	-24.14288	-18.95991
C	17.23188	-25.10663	-21.15496
C	18.09725	-23.01364	-19.44614
C	17.90176	-23.98922	-21.67416
C	18.33869	-22.95600	-20.82924
H	16.92198	-25.91648	-21.82231
H	18.09664	-23.92797	-22.75126
H	18.86798	-22.09880	-21.25649
O	17.17520	-24.25417	-17.60816
C	18.47133	-21.92122	-18.42951
C	17.19531	-21.07917	-18.13680
H	17.41903	-20.29375	-17.39329
H	16.83229	-20.60165	-19.06436
H	16.38593	-21.71231	-17.73537
C	19.57751	-20.99608	-18.95700
H	19.81361	-20.21309	-18.21788
H	20.50194	-21.55534	-19.18204
H	19.24970	-20.47856	-19.87397
C	15.71603	-25.46772	-15.02008
C	14.68095	-26.42334	-15.03081
C	15.41101	-24.12430	-14.73312
C	13.36754	-26.04611	-14.72848
C	14.09056	-23.74593	-14.44677
C	13.06873	-24.70638	-14.43636
H	14.91180	-27.46387	-15.28875
H	16.20876	-23.37316	-14.72506
H	12.57009	-26.79690	-14.74297
H	13.86110	-22.69652	-14.22435
H	12.03749	-24.40964	-14.21145
C	14.58533	-26.30888	-18.49761
C	14.11750	-24.98868	-18.36763
C	13.70715	-27.37889	-18.22060
C	12.79174	-24.74469	-17.98057
C	12.37865	-27.13130	-17.85633
C	11.91597	-25.81109	-17.73494
H	14.78495	-24.14690	-18.57266
H	14.06672	-28.41153	-18.30788

H	12.44250	-23.71118	-17.87073
H	11.70347	-27.97233	-17.65749
H	10.87951	-25.61468	-17.43626
C	17.99006	-26.62350	-13.69988
C	19.04046	-27.55900	-13.63346
C	17.43324	-26.12252	-12.50651
C	19.53915	-27.97552	-12.39101
C	17.92643	-26.54954	-11.26516
C	18.98174	-27.47346	-11.20485
H	19.45387	-27.96209	-14.56600
H	16.61066	-25.39991	-12.55006
H	20.35963	-28.70221	-12.34925
H	17.48411	-26.15918	-10.34091
H	19.36448	-27.80741	-10.23332
C	16.04430	-27.84327	-20.40687
C	16.96094	-28.88308	-20.64735
C	14.96525	-27.65303	-21.29540
C	16.80796	-29.71878	-21.76477
C	14.81036	-28.49203	-22.40770
C	15.73270	-29.52555	-22.64547
H	17.78786	-29.03303	-19.94230
H	14.24682	-26.84631	-21.11176
H	17.52712	-30.52787	-21.94121
H	13.96665	-28.33976	-23.09214
H	15.60943	-30.18185	-23.51563
C	18.88268	-29.07631	-17.27402
O	19.58816	-29.99974	-17.30194

$Pd(PMe_2Ph)_2(CO)$ ($Pd(\mathbf{4})_2(CO)$)

Pd	0.04662	-0.81206	-1.67522
P	-1.89874	-0.05305	-0.57770
P	1.87456	0.11185	-0.49066
C	-1.89773	-0.05086	1.26712
C	-2.53038	-1.06475	2.01521
C	-1.14643	0.92484	1.95768
C	-2.42373	-1.09331	3.41450
H	-3.11025	-1.84436	1.50936
C	-1.04704	0.90071	3.35294
H	-0.61879	1.70404	1.39722
C	-1.68510	-0.11058	4.08839
H	-2.92319	-1.89048	3.97906
H	-0.45116	1.66296	3.86806
H	-1.59563	-0.13768	5.18114
C	1.88909	-0.29579	1.31292
C	1.18997	-1.44856	1.72435
C	2.55825	0.47823	2.28048
C	1.16533	-1.82385	3.07334
H	0.64450	-2.03515	0.97425
C	2.52297	0.10955	3.63374
H	3.10908	1.37896	1.98562
C	1.82875	-1.04306	4.03165
H	0.60893	-2.71641	3.38082
H	3.03930	0.72547	4.38011
H	1.79684	-1.32738	5.09040
C	3.60542	-0.36511	-0.97690
H	4.36345	0.12160	-0.33631
H	3.70006	-1.46058	-0.89163
H	3.76675	-0.08127	-2.03147
C	2.01030	1.96417	-0.53041
H	2.92399	2.34341	-0.03951
H	2.01965	2.27929	-1.58784
H	1.12494	2.40896	-0.04644
C	-3.50035	-0.89461	-0.98817
H	-3.67379	-0.75606	-2.06923
H	-3.40782	-1.97784	-0.80305
H	-4.35249	-0.48673	-0.41511
C	-2.33184	1.71224	-0.97006
H	-2.50563	1.78512	-2.05746
H	-3.23233	2.04219	-0.42249
H	-1.48621	2.37291	-0.71880
C	0.05995	-1.92669	-3.23076
O	0.04014	-2.59381	-4.18233

$Pd(PEt_2Ph)_2(CO)$ ($Pd(\mathbf{2})_2(CO)$)

Pd	-0.02836	-0.50874	-1.81045
P	-2.02247	-0.10729	-0.60515
P	1.88534	-0.18544	-0.45699
C	-1.94007	0.18703	1.21911
C	-2.71022	-0.50016	2.17682
C	-0.99319	1.13140	1.66873
C	-2.52724	-0.25752	3.54792
H	-3.45705	-1.23784	1.86374
C	-0.82296	1.38819	3.03318
H	-0.36459	1.64671	0.93232
C	-1.58469	0.68531	3.98015
H	-3.12654	-0.81183	4.28040
H	-0.07392	2.11826	3.36242
H	-1.43556	0.86647	5.05128
C	1.75575	-0.64845	1.32766
C	0.82237	-1.64669	1.67312
C	2.51404	-0.04394	2.34932
C	0.64613	-2.03031	3.00851
H	0.21275	-2.09788	0.88107
C	2.32824	-0.41814	3.68881
H	3.24897	0.73218	2.10729
C	1.39403	-1.41062	4.02105
H	-0.09287	-2.79956	3.26215
H	2.91440	0.07017	4.47669
H	1.24349	-1.69567	5.06913
C	3.40337	-1.15441	-0.96486
H	4.21886	-0.93953	-0.24737
H	3.70199	-0.74834	-1.94930
C	2.55087	1.55846	-0.41168
H	3.58256	1.54877	-0.01258
H	1.92838	2.11561	0.31243
C	-3.27726	-1.46977	-0.79141
H	-3.60553	-1.40122	-1.84516
H	-4.16695	-1.27631	-0.16374
C	-2.95695	1.41072	-1.17657
H	-3.11421	1.26334	-2.26202
H	-2.23649	2.24497	-1.08332
C	-0.00197	-1.02618	-3.65086
O	0.01534	-1.30758	-4.77924
C	2.49981	2.22570	-1.79048
H	3.10656	1.67519	-2.53072
H	1.46379	2.25237	-2.17392
H	2.88428	3.26084	-1.74257
C	3.12603	-2.65491	-1.06264
H	4.01998	-3.20271	-1.41253
H	2.83719	-3.07266	-0.08157
H	2.29987	-2.85216	-1.77049
C	-2.67836	-2.85235	-0.51315
H	-3.42157	-3.65118	-0.68992
H	-1.80899	-3.03406	-1.17004
H	-2.33044	-2.94201	0.53143
C	-4.26299	1.72897	-0.44825
H	-5.00977	0.92437	-0.57567
H	-4.09393	1.86301	0.63500
H	-4.71169	2.66063	-0.84034

$Pd(PEt_2Ph)_2(CO)$ ($Pd(\mathbf{2})_2(CO)$)

Pd	-0.02836	-0.50874	-1.81045
P	-2.02247	-0.10729	-0.60515
P	1.88534	-0.18544	-0.45699
C	-1.94007	0.18703	1.21911
C	-2.71022	-0.50016	2.17682
C	-0.99319	1.13140	1.66873
C	-2.52724	-0.25752	3.54792
H	-3.45705	-1.23784	1.86374
C	-0.82296	1.38819	3.03318
H	-0.36459	1.64671	0.93232
C	-1.58469	0.68531	3.98015
H	-3.12654	-0.81183	4.28040
H	-0.07392	2.11826	3.36242
H	-1.43556	0.86647	5.05128
C	1.75575	-0.64845	1.32766
C	0.82237	-1.64669	1.67312
C	2.51404	-0.04394	2.34932
C	0.64613	-2.03031	3.00851
H	0.21275	-2.09788	0.88107
C	2.32824	-0.41814	3.68881
H	3.24897	0.73218	2.10729
C	1.39403	-1.41062	4.02105
H	-0.09287	-2.79956	3.26215
H	2.91440	0.07017	4.47669
H	1.24349	-1.69567	5.06913
C	3.40337	-1.15441	-0.96486
H	4.21886	-0.93953	-0.24737
H	3.70199	-0.74834	-1.94930
C	2.55087	1.55846	-0.41168
H	3.58256	1.54877	-0.01258
H	1.92838	2.11561	0.31243
C	-3.27726	-1.46977	-0.79141
H	-3.60553	-1.40122	-1.84516
H	-4.16695	-1.27631	-0.16374
C	-2.95695	1.41072	-1.17657
H	-3.11421	1.26334	-2.26202
H	-2.23649	2.24497	-1.08332
C	-0.00197	-1.02618	-3.65086
O	0.01534	-1.30758	-4.77924
C	2.49981	2.22570	-1.79048
H	3.10656	1.67519	-2.53072
H	1.46379	2.25237	-2.17392
H	2.88428	3.26084	-1.74257
C	3.12603	-2.65491	-1.06264
H	4.01998	-3.20271	-1.41253
H	2.83719	-3.07266	-0.08157
H	2.29987	-2.85216	-1.77049
C	-2.67836	-2.85235	-0.51315
H	-3.42157	-3.65118	-0.68992
H	-1.80899	-3.03406	-1.17004
H	-2.33044	-2.94201	0.53143
C	-4.26299	1.72897	-0.44825
H	-5.00977	0.92437	-0.57567
H	-4.09393	1.86301	0.63500
H	-4.71169	2.66063	-0.84034

$Pd(PEt_3)_2(CO)$ (Pd(**1**)$_2$(CO))

Pd	0.10584	-1.36451	-1.54849
P	-1.90235	-0.46267	-0.67027
P	1.89074	-0.25516	-0.47792
C	3.62841	-0.87222	-0.80154
H	4.33179	-0.34666	-0.12594
H	3.86095	-0.53975	-1.83103
C	2.06657	1.54183	-0.95892
H	3.02171	1.93603	-0.55966
H	1.25437	2.08142	-0.43760
C	-3.45176	-0.72407	-1.70259
H	-3.26026	-1.65835	-2.25945
H	-3.42795	0.08279	-2.46151
C	-1.88367	1.37661	-0.31596
H	-1.48858	1.84256	-1.23820
H	-1.08269	1.49261	0.43988
C	0.24417	-2.68205	-2.92776
O	0.34183	-3.44000	-3.80540
C	1.95734	1.75897	-2.47032
H	2.76007	1.23450	-3.01881
H	0.99595	1.36771	-2.84971
H	2.02429	2.83223	-2.72634
C	3.78013	-2.39161	-0.70641
H	4.78531	-2.71104	-1.03726
H	3.64077	-2.75495	0.32668
H	3.03004	-2.89484	-1.34259
C	-4.81529	-0.79250	-1.00716
H	-5.62277	-0.90132	-1.75473
H	-4.88054	-1.66340	-0.33134
H	-5.03519	0.10748	-0.40962
C	-3.16286	2.08502	0.13639
H	-3.93447	2.06912	-0.65326
H	-3.59457	1.62162	1.04065
H	-2.95784	3.14572	0.37337
C	1.82996	-0.09722	1.38538
H	2.76204	0.37983	1.74861
H	1.00856	0.61853	1.58257
C	1.55678	-1.41447	2.11591
H	2.41084	-2.10984	2.04300
H	0.67859	-1.92559	1.68228
H	1.36016	-1.24108	3.18996
C	-2.33376	-1.18637	0.99467
H	-1.56928	-0.79300	1.69043
H	-3.31176	-0.80889	1.34459
C	-2.30448	-2.71710	0.98186
H	-1.32398	-3.08689	0.63281
H	-3.06825	-3.13181	0.30070
H	-2.49405	-3.12928	1.99004

$Pd(PMe_3)_2(CO)$ (Pd(**3**)$_2$(CO))

Pd	-0.05427	1.17105	-1.61266
P	-1.89423	0.07145	-0.62267
P	1.87985	0.23468	-0.63146
C	1.94574	-1.61311	-0.43852
H	2.88974	-1.95167	0.02823
H	1.84386	-2.08456	-1.43102
C	3.52839	0.55554	-1.42445
H	4.36237	0.08451	-0.87066
H	3.68660	1.64633	-1.47878
C	-2.17235	-1.66485	-1.22115
H	-1.27175	-2.26961	-1.01671
H	-2.32976	-1.64269	-2.31315
C	-3.58937	0.79732	-0.84467
H	-3.79728	0.88354	-1.92488
H	-3.59773	1.81400	-0.41581
C	-0.12889	2.53715	-2.95088
O	-0.17819	3.36151	-3.76921
C	2.21179	0.78374	1.11135
H	3.12628	0.31754	1.52290
H	2.32235	1.88167	1.12346
C	-1.87098	-0.18911	1.21841
H	-1.78972	0.78996	1.72079
H	-2.77940	-0.70760	1.57866
H	-3.04710	-2.13341	-0.73313
H	-4.37613	0.18795	-0.36108
H	-0.98301	-0.78743	1.48625
H	1.34907	0.51910	1.74724
H	3.50677	0.16453	-2.45612
H	1.09605	-1.94040	0.18512

$Pd(PPh_3)_2(CO)$ ($Pd(\mathbf{5})_2(CO)$)

Pd	-0.07742	-1.28611	-0.98394
P	1.89542	-0.12814	-0.38547
P	-1.91854	-0.19847	0.02125
C	1.84305	1.70695	-0.20136
C	2.45468	2.56761	-1.13344
C	1.06904	2.26752	0.83887
C	2.31296	3.95951	-1.01463
H	3.04774	2.15023	-1.95449
C	0.94867	3.65478	0.96710
H	0.55639	1.61331	1.55153
C	1.56876	4.50729	0.03927
H	2.79490	4.61621	-1.74927
H	0.34681	4.07215	1.78196
H	1.45959	5.59470	0.13250
C	3.38383	-0.37522	-1.45778
C	3.21507	-0.94137	-2.73412
C	4.67430	-0.00215	-1.03010
C	4.32112	-1.12539	-3.57885
H	2.21046	-1.24689	-3.05167
C	5.77794	-0.19059	-1.87223
H	4.81312	0.43634	-0.03590
C	5.60276	-0.75130	-3.14898
H	4.18028	-1.56795	-4.57184
H	6.77923	0.10000	-1.53220
H	6.46819	-0.90031	-3.80590
C	2.50682	-0.70721	1.26301
C	2.34250	-2.07780	1.55846
C	3.08510	0.13761	2.23019
C	2.74305	-2.59085	2.79919
H	1.87554	-2.73209	0.81148
C	3.47798	-0.37714	3.47571
H	3.21711	1.20317	2.01606
C	3.30660	-1.73985	3.76444
H	2.60177	-3.65594	3.01835
H	3.91868	0.29235	4.22445
H	3.60974	-2.13767	4.74009
C	-2.07867	-0.42226	1.84968
C	-3.32093	-0.40853	2.51535
C	-0.90061	-0.61537	2.59929
C	-3.37597	-0.56570	3.90852
H	-4.24561	-0.28022	1.94133
C	-0.95662	-0.76770	3.99146
H	0.06413	-0.66706	2.08552
C	-2.19616	-0.74177	4.64972
H	-4.34743	-0.55481	4.41825
H	-0.02782	-0.92165	4.55308
H	-2.24595	-0.87169	5.73775
C	-3.59969	-0.69416	-0.57178
C	-3.84756	-2.07427	-0.72189
C	-4.62111	0.22566	-0.87630
C	-5.10313	-2.52692	-1.14563
H	-3.03964	-2.78818	-0.51762
C	-5.87474	-0.23069	-1.31575
H	-4.43816	1.30105	-0.77372
C	-6.12041	-1.60507	-1.44615
H	-5.28541	-3.60295	-1.25665
H	-6.66177	0.49373	-1.55821
H	-7.09891	-1.95967	-1.79125
C	-1.94829	1.63516	-0.19122
C	-1.38981	2.15179	-1.37791
C	-2.49452	2.52037	0.75721
C	-1.39457	3.53023	-1.62197
H	-0.93186	1.46116	-2.09705
C	-2.49339	3.90204	0.51302
H	-2.91489	2.12864	1.68963
C	-1.94816	4.40826	-0.67680
H	-0.94590	3.92257	-2.54155
H	-2.91506	4.58653	1.25914
H	-1.93978	5.48941	-0.86108
C	-0.14708	-2.86102	-2.07104
O	-0.18428	-3.83387	-2.70478

$Pd(P(OMe)_3)_2(CO)$ ($Pd(\mathbf{6})_2(CO)$)

Pd	0.95693	0.34906	0.35313
P	-0.26746	1.47622	-1.26357
P	0.71859	-1.92488	-0.03442
O	-0.80378	-2.36598	-0.53334
O	1.01507	-3.04222	1.14617
O	1.70405	-2.57614	-1.21009
O	-0.16396	0.78415	-2.77155
O	-1.91069	1.58918	-1.01403
O	0.01499	3.06971	-1.59420
C	2.00814	1.15547	1.76156
O	2.64256	1.63255	2.60394
C	-2.59264	0.50125	-0.38179
H	-2.07036	0.17808	0.53837
H	-3.60173	0.86251	-0.12294
H	-2.66864	-0.36950	-1.05764
C	-0.79043	1.37071	-3.91401
H	-0.30277	2.32569	-4.18417
H	-0.67963	0.65891	-4.74970
H	-1.86679	1.55043	-3.73120
C	-0.96503	4.10260	-1.69557
H	-0.41200	5.05463	-1.76267
H	-1.59457	3.98766	-2.59886
H	-1.62448	4.12193	-0.80963
C	-1.14929	-3.73380	-0.76137
H	-0.44879	-4.20955	-1.47396
H	-1.14663	-4.30393	0.18628
H	-2.16586	-3.74655	-1.18927
C	1.89012	-4.16532	1.04277
H	1.97564	-4.59207	2.05652
H	1.49017	-4.93621	0.35653
H	2.89192	-3.86699	0.68578
C	2.01126	-1.78502	-2.36234
H	2.41785	-0.79673	-2.07477
H	2.77019	-2.34084	-2.93863
H	1.11339	-1.61781	-2.98437

$Pd(P(OPh)_3)_2(CO)$ ($Pd(\mathbf{7})_2(CO)$)

Pd	0.33988	-0.10456	-0.91959
P	-1.38658	1.04370	-1.89830
C	-2.19986	1.44170	0.55124
C	-2.38623	-0.49428	-3.84959
C	0.06981	3.08115	-2.86587
C	-1.64199	0.74224	3.20396
C	-1.52327	2.07799	2.78643
C	-1.79673	2.43334	1.45665
C	-2.35518	0.10828	0.96773
C	-2.06644	-0.23835	2.29515
H	-1.40090	0.46357	4.23578
H	-1.19691	2.84876	3.49439
H	-1.68473	3.46399	1.10384
H	-2.70316	-0.63904	0.24834
H	-2.15480	-1.28218	2.61089
C	-2.06079	-2.32861	-5.94700
C	-1.02068	-1.45278	-5.60987
C	-1.17969	-0.52344	-4.57003
C	-3.43330	-1.37116	-4.17922

C	-3.26887	-2.27755	-5.23136
H	-1.93162	-3.05119	-6.76058
H	-0.07279	-1.48196	-6.16017
H	-0.37795	0.18170	-4.33342
H	-4.35619	-1.33545	-3.59296
H	-4.08463	-2.96615	-5.47752
P	0.78274	-2.28544	-1.54011
C	0.70044	-3.17303	0.99169
C	3.07160	-1.26242	-2.21068
C	-1.27211	-3.68367	-2.53906
C	0.30560	-2.64813	3.71484
C	-0.58037	-3.49133	3.02349
C	-0.39475	-3.74684	1.65726
C	1.60265	-2.34340	1.67683
C	1.39295	-2.07566	3.03760
H	0.14837	-2.43833	4.77920
H	-1.42939	-3.94652	3.54770
H	-1.08094	-4.38641	1.09197
H	2.45491	-1.91587	1.14126
H	2.08765	-1.41394	3.56800
C	4.56783	1.08580	-1.94692
C	3.46343	1.05406	-2.81156
C	2.71492	-0.12356	-2.95204
C	4.18228	-1.24677	-1.35659
C	4.93310	-0.06716	-1.23276
H	5.14243	2.01156	-1.82852
H	3.16464	1.94993	-3.36344
H	1.85419	-0.17209	-3.62561
H	4.43022	-2.14965	-0.78884
H	5.79872	-0.04634	-0.56019
C	2.40767	4.63167	-2.85493
C	1.85392	4.19696	-4.07071
C	0.69182	3.41290	-4.08118
C	0.60708	3.52247	-1.64448
C	1.78194	4.28826	-1.64701
H	3.32269	5.23471	-2.85029
H	2.33483	4.46061	-5.01973
H	0.24847	3.05493	-5.01597
H	0.10947	3.26799	-0.70602
H	2.20515	4.62051	-0.69227
C	-3.90690	-4.65662	-2.41274
C	-3.47120	-3.67653	-1.50971
C	-2.15733	-3.18954	-1.56373
C	-1.70057	-4.66578	-3.44654
C	-3.01304	-5.14829	-3.37790
H	-4.93323	-5.03704	-2.36405
H	-4.15687	-3.27897	-0.75155
H	-1.82980	-2.42114	-0.85672
H	-0.99337	-5.02939	-4.19820
H	-3.34017	-5.91540	-4.09030
O	0.02875	-3.24708	-2.67499
O	0.87547	-3.46752	-0.34968
O	2.27740	-2.40669	-2.27205
O	-2.61772	0.37990	-2.80254
O	-2.38825	1.78853	-0.78199
O	-1.08818	2.32875	-2.93194
C	1.42175	0.78155	0.43527
O	2.05857	1.33219	1.22590

$Pd(PCl_2(OEt))_2(CO)$ (Pd(**27**)$_2$(CO))

Pd	0.87491	0.29110	0.59384
P	-0.40987	1.38524	-0.97429
P	0.99632	-1.87771	-0.17604
C	1.66115	1.04135	2.21801
O	2.10851	1.48454	3.18006
Cl	-2.43631	0.90863	-0.82334
Cl	-0.01812	0.96929	-2.98512
Cl	-0.77934	-2.72250	-0.88219
Cl	1.69839	-3.42956	1.04684
Cl	2.25405	-2.06968	-1.83080
Cl	-0.48985	3.48010	-1.02892

$Pd(PCl_3)_2(CO)$ (Pd(**9**)$_2$(CO))

Pd	0.87491	0.29110	0.59384
P	-0.40987	1.38524	-0.97429
P	0.99632	-1.87771	-0.17604
C	1.66115	1.04135	2.21801
O	2.10851	1.48454	3.18006
Cl	-2.43631	0.90863	-0.82334
Cl	-0.01812	0.96929	-2.98512
Cl	-0.77934	-2.72250	-0.88219
Cl	1.69839	-3.42956	1.04684
Cl	2.25405	-2.06968	-1.83080
Cl	-0.48985	3.48010	-1.02892

$Pd(PF_3)_2(CO)$ (Pd(**10**)$_2$(CO))

Pd	0.72692	0.17896	0.39357
P	-0.67602	1.38085	-0.93887
P	1.18494	-1.96676	-0.22082
C	1.52283	0.97704	2.00655
O	1.99381	1.44288	2.94514
F	-2.16484	1.58357	-0.37098
F	-0.33083	2.91549	-1.27180
F	1.44836	-3.09367	0.89613
F	-1.06244	0.93410	-2.43619
F	0.19352	-2.83288	-1.14608
F	2.52904	-2.16094	-1.07962

$Pd(P(CF_3)F_2)_2(CO)$ (Pd(**11**)$_2$(CO))

Pd	1.36847	0.29220	0.32372
P	-0.67584	0.93565	-0.41004
P	1.69703	-1.90902	-0.10051
C	2.68327	1.51218	1.15731
O	3.44727	2.22377	1.63577
F	-2.01764	0.28610	0.21149
F	-1.14705	2.48598	-0.44341
F	1.63662	-2.42017	-1.63173
F	2.99428	-2.75153	0.38237
C	-1.04716	0.56184	-2.25379
F	-2.22862	1.03769	-2.66672
F	-0.06830	1.11444	-3.00164
F	-1.02684	-0.77190	-2.44486
C	0.38451	-3.10207	0.62778
F	-0.81999	-2.80268	0.10231
F	0.64738	-4.39426	0.39586
F	0.32889	-2.89997	1.96162
